# 1133. Opportunities for Antibiotic Discontinuation and De-escalation after Discharge from the Emergency Department in Pediatric Patients with UTI

**DOI:** 10.1093/ofid/ofab466.1326

**Published:** 2021-12-04

**Authors:** Stephanie Hawkins, Patrick Gavigan, Jessica E Ericson, George McSherry

**Affiliations:** 1 Penn State College of Medicine, Hershey, Pennsylvania; 2 Penn State Children’s Hospital, Hummelstown, Pennsylvania; 3 Penn State Hershey, Hershey, PA

## Abstract

**Background:**

In children, urinary tract infection (UTI) represents one of the most common indications for antibiotics. While previous data has demonstrated high rates of misdiagnosis and inconsistencies with empiric antibiotics, the impact and opportunities for antibiotic reduction once final culture and susceptibility data are available, particularly in pediatric patients seen in the emergency department (ED), is unknown.

**Methods:**

This was a retrospective study conducted over a period of 18-months, which included subjects less than 18 years of age who were discharged from the ED with a diagnosis of UTI. Episodes in which urine cultures were negative or grew only mixed urogenital flora were considered possible for discontinuation. De-escalation was considered possible in episodes in which identified bacteria were susceptible to more narrow spectrum agents than the prescribed empiric antibiotic. Rates of discontinuation and de-escalation were calculated as proportions, and excess days of therapy were described. Subjects whose empiric antibiotics were active against isolated bacteria were compared to those with bacteria resistant to empiric therapy.

**Results:**

A total of 87 episodes of UTI were identified. Pathogenic bacteria were isolated in 51 (59%) of the 78 episodes in which urine cultures were sent, most commonly *Escherichia coli* (84%). Empiric antibiotic therapy and duration varied and were active against isolated bacteria in 39 (76%) of the episodes. Subjects whose antibiotics were inactive were more likely to be Hispanic and receive cephalexin [Table 3]. Antibiotics were discontinued in 3 of the 27 possible episodes (11%), resulting in 127 extra antibiotic days, median of 6 (IQR=10 days) days per episode. In 20 episodes there was an opportunity for de-escalation, but it was never attempted, leading to 131 extra days of broad-spectrum antibiotics (median 7.5 days, IQR=3).

Table 1. Microbiology and resistance profile of bacteria isolated from urine cultures

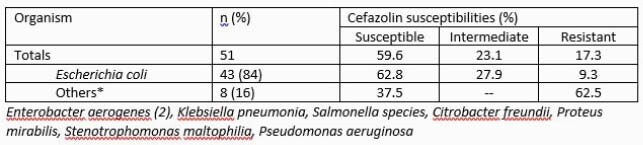

Table 2. Empiric antibiotic regimens, including type of antibiotic and duration

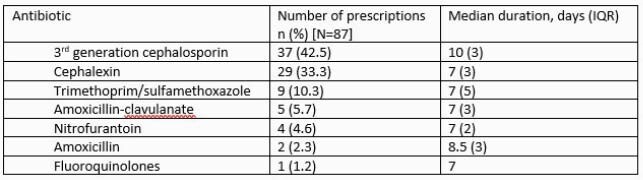

Table 3. Comparison of episodes in which empiric antibiotics were active against isolated bacteria versus those in which empiric antibiotics were inactive

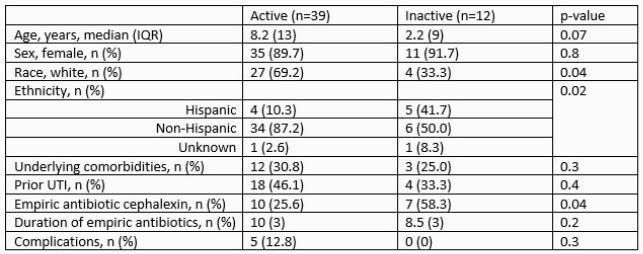

**Conclusion:**

Antibiotics are rarely adjusted after discharge from the ED. Lack of adjustment results in unnecessary total and broad-spectrum antibiotic exposures. Initiatives designed to improve antibiotic use post-discharge could result in significant decreases in unnecessary antibiotics, and ultimately reduced rates of antibiotic resistance.

**Disclosures:**

**All Authors**: No reported disclosures

